# Administration and Therapeutic Drug Monitoring of *β-lactams* and Vancomycin in Critical Care Units in Colombia: The ANTIBIOCOL Study

**DOI:** 10.3390/pharmaceutics13101577

**Published:** 2021-09-28

**Authors:** Yuli V. Fuentes, Jhosep Blanco, Diana Marcela Díaz-Quijano, Sharon Lechtig-Wasserman, Hans Liebisch-Rey, Nicolas Díaz-Pinilla, Peter Vergara-Ramirez, Rosa-Helena Bustos

**Affiliations:** 1 Master’s Student in Epidemiology, Faculty of Medicine, Universidad de La Sabana, Chía 140013, Colombia; yulyfuba@unisabana.edu.co; 2 Evidence-Based Therapeutics Group, Department of Clinical Pharmacology, Faculty of Medicine, Universidad de La Sabana and Clínica Universidad de La Sabana, Chía 140013, Colombia; jhosepblme@unisabana.edu.co (J.B.); sharonlele@unisabana.edu.co (S.L.-W.); hanslire@unisabana.edu.co (H.L.-R.); nicolasdipi@unisabana.edu.co (N.D.-P.); peter.vergara@unisabana.edu.co (P.V.-R.); 3 Health Research Group, Department of Epidemiology, Faculty of Medicine, Universidad de La Sabana, Chía 140013, Colombia; diana.diaz1@unisabana.edu.co

**Keywords:** drug monitoring, antimicrobial stewardship, critical care, vancomycin, *beta-lactams*, health care surveys, infusions, intravenous

## Abstract

Therapeutic drug monitoring (TDM) and continuous infusion strategies are effective interventions in clinical practice, but these practices are still largely unknown in Colombia, especially in the critical care setting. This study aims to describe the practices involved in the administration and TDM of *β-lactams* and vancomycin reported by specialists in critical care in Colombia and to explore the factors that are related to the use of extended infusion. An online nationwide survey was applied to 153 specialists, who were selected randomly. A descriptive, bivariate analysis and a logistic regression model were undertaken. In total, 88.9% of the specialists reported TDM availability and 21.57% reported access to results within 6 h. TDM was available mainly for vancomycin. We found that 85.62% of the intensivists had some type of institutional protocol; however, only 39.22% had a complete and socialized protocol. The odds of preferring extended infusions among those who did not have institutional protocols were 80% lower than those with complete protocols, OR 0.2 (95% CI: 0.06−0.61). The most important perceived barriers to performing continuous infusions and TDM were the lack of training and technologies. This pioneering study in Colombia could impact the quality of care and outcomes of critically ill patients in relation to the threat of antimicrobial resistance.

## 1. Introduction

Antibiotic therapeutic drug monitoring (TDM) refers to the individualization of drug dosages by maintaining plasma drug concentrations within a targeted therapeutic range, which is based on pharmacokinetic (PK) and pharmacodynamic (PD) properties [1,2,3]. TDM could function as a tool to minimize toxicity, improve clinical efficacy and optimize antibiotic stewardship, especially in critical care settings [4,5], because this subset of patients have various PK changes, leading to either a decrease in clinical effectiveness or a higher toxicity risk [6].

Vancomycin and *β-lactams* are the antibiotics most frequently used and measured in plasma in the intensive care unit (ICU) setting, because of their antimicrobial spectrum and their PK/PD characteristics [7,8]. Vancomycin TDM and its use in continuous or prolonged infusion (PI) could have a beneficial effect in reaching PK targets and reducing nephrotoxicity and renal replacement therapy [9,10]. The area under the curve-to-minimum inhibitory concentration (AUC/MIC) ratio has been identified as the most appropriate pharmacokinetic/pharmacodynamic (PK/PD) target for vancomycin [9]. Rybak et al. recognized that continuous infusion may be a reasonable alternative to conventional intermittent infusion dosing when the AUC target cannot be achieved [9].

With reference to β-lactam’s TDM literature status, studies have shown positive results in terms of clinical cure rates (the reduction of procalcitonin in patients with TDM follow-up), a shorter mean duration of invasive ventilation, the achievement of PK parameters and an easier dose guidance process, particularly in critically ill patients or patients undergoing renal replacement therapy [7,11,12,13,14,15,16]. More clinical studies should be developed to demonstrate the conclusive benefits of this practice. Furthermore, a recent meta-analysis showed that the continuous infusion of *β-lactams* increased the achievement of the target plasma concentrations and significantly improved the clinical cure rate [17].

Clinical practice guidelines recommend the use of extended or continuous infusions and the use of TDM with early obtainment of plasma concentration results between 24−48 h after the start of treatment with vancomycin or *β-lactams* [7,10,18,19,20]. The literature recommends the use of specialized software, as well as the reporting of the minimum inhibitory concentration (MIC) of the isolated microorganism, to generate adequate PK modeling [21,22,23]. Likewise, TDM and antimicrobial stewardship should be guided by an infectious disease specialist, because evidence shows that there is a positive impact on clinical outcomes, such as a reduction in mortality, a reduced length of stay, lower readmissions and costs, and a more rational use of antibiotics, when they support decision-making [24]. Among the interventions carried out in clinical practice, guiding therapy based on antibiotic institutional protocols has shown improvements in clinical outcomes, as well as reductions in the use of institutional resources, and increasing compliance with antibiotic stewardship programs [25,26,27,28]. Nonetheless, previous studies in developed countries have reported barriers such as the lack of protocols and pharmaceutical information, and the unavailability of required technologies [29,30,31,32,33].

Antibiotic TDM is a novel intervention which is expanding and gaining a place in clinical practice, especially in the critically ill patient context [5]. Several studies about the TDM practices (from Europe, New Zealand, and North América) have been published, with a high variability in practices, availability, and obstacles [29,30,31,32,33]. TDM practices and barriers in Colombia and Latin America have been explored in only one study, which used a non-representative sample [32]. Knowledge about TDM practices and barriers to its implementation is a key factor in the advancement of personalized medicine, with benefits associated with this intervention. Therefore, the main objective of this study was to describe the management practices and therapeutic monitoring of β-lactam and vancomycin, as well as to explore factors associated with the use of continuous or PI, as reported by critical care specialists in Colombia.

## 2. Materials and Methods

A descriptive cross-sectional study with an analytical component was conducted. A national survey was designed based on 12 general questions from the ANTIBIOPERF study [30] (48% of the survey) and an additional 13 specific questions were elaborated by a multidisciplinary team of infectious disease specialists, intensivists, and epidemiologists according to the Colombian context and the objectives of this study. The appearance validity of the questionnaire was evaluated through an initial pilot test with 10 intensivists who shared comments and suggestions based on the understandability of the questions, the relevance of the questions, the response time, and the ease of browsing inside the virtual platform. The survey was adjusted according to the suggestions made and then subsequently applied to a sample of professionals in charge of adult critical care units across the country. Through the Colombian Association of Critical Medicine and Intensive Care (AMCI), the information was collected electronically by sending the survey via email to the selected specialists in the sample between October 2020 and April 2021.

The questionnaire included 25 multiple-choice questions divided into 4 sections: sociodemographic information, clinical practice, clinical knowledge, and perception. The antibiotics chosen for the study were oxacillin, ampicillin-ampicillin/sulbactam, cefazolin, cefuroxime, ceftriaxone, ceftazidime, cefepime, piperacillin-tazobactam, meropenem, ertapenem, doripenem, and vancomycin. The types of administration were considered continuous infusions if done over 24 h (replacing the solution every 8−12 h), PI if between 2 to 4 h, and intermittent infusions if less than 30 min. Each participant provided only their email address as the only personal data collected and they all voluntarily provided informed consent for the completion of the survey. To improve the response rate, participants were informed that they would enter a sweepstakes for 3 gift vouchers after the end of the study to avoid bias in the results. The resources for these incentives were provided by the researchers and do not represent any conflict of interest. This study was approved by the research and ethics subcommittee (Acta 514, 25 September 2020) of the Universidad de La Sabana.

### 2.1. Study Population, Recruitment and Sample

The target population was professionals in critical medicine and intensive care who work in one or more adult critical care units in Colombia, public or private, regardless of their first specialty. A single response per specialist was considered, based on the institution where they work most of the time. In August 2020, there were 255 specialists in critical medicine and adult intensive care throughout the country, who were registered, certified, and validated by the AMCI. From this sampling frame, for a confidence level of 95%, a margin of error of 5% and considering a prevalence of 0.5, the survey was carried out with 153 intensivists, chosen through a probabilistic sampling of a simple random design with a negative coordinated method. The survey was disseminated online through the SurveyMonkey^®^ electronic platform (the instrument is available in the Supplementary Information section), and monthly reminders were emailed. Subsequently, it was verified that participants who answered the survey were the ones selected by sampling; otherwise, the answers were not considered in the statistical analysis.

### 2.2. Statistical Analysis

Data were exported from the SurveyMonkey^®^ electronic platform to a Microsoft Excel^®^ file and subsequently analyzed using the statistical software Rstudio^®^ version 3.6.1 (R Development Core Team, Vienna, Austria.)

A variable of interest, entitled “approach to clinical cases”, was created based on the seven questions of the clinical knowledge section of the survey (question 16–22). This variable was calculated by assigning a value of 1.5 to a correct answer for question 16–21 and a value of 0.1 to a correct answer for each of the ten vignettes of the 22 questions, giving a total score of 1 for this question. The last question (question 22) had less value because it was related to non-clinical issues that do not usually belong to the intensivist’s field of experience. The final continuous score was between 0 and 10. A dichotomous outcome variable was created based on questions 17, 18, and 20, according to the respondent’s preference for using continuous infusion or PI. If a participant reported the use of continuous or prolonged infusions in 2/3 or 3/3 questions, they were recorded as having a preference for prolonged infusions; otherwise, they were categorized as having a preference for intermittent infusions. Correct answers were based on the literature [9,34].

Initially, a descriptive analysis of the information was performed using means and standard deviations for continuous quantitative variables with a normal distribution, and medians with interquartile ranges for continuous quantitative variables with another distribution. Categorical variables were described with absolute and relative frequencies. The association between administration practices and TDM was explored with the “approach to clinical cases” variable (Mann-Whitney U test and Kruskal-Wallis test) and a variable based on the preference for the use of continuous or prolonged infusions (Mann-Whitney U test and Chi-squared test). A logistic regression model was used to explore associations in which the preference to use continuous or PI was included as an outcome variable. Sociodemographic and clinical practice data were the independent variables. Questions about perception were excluded from the model due to their absence of biomedical and epidemiological significance [35]. Variables were adjusted to select the model that best explained the response variable (*p*-value < 0.05 in Wald tests). Odds ratios (ORs) were calculated based on the the exponentials of the coefficients obtained by the final model. A significance level of 0.05 and a confidence level of 95% were chosen.

## 3. Results

### 3.1. Sociodemographic Information

Of the 255 registered intensivists, 153 were selected randomly, with a response rate of 100%. Participants had a median age of 43.11 years with a median of 10 years of experience; 67.97% were men; 53.59% worked in a university hospital, most of them in a medical ICU. Only 30.72% had received formal education about the use of antimicrobial therapies and 19.60% (30/153) did not report any type of training in this area. The participation by cities was distributed as follows: Bogotá 31.37% (48/153); Medellin 11.11% (17/153); Manizales and Pereira 7.19% each (11/153); Cali 5.88% (9/153); Cartagena and Cúcuta 3.92% each (6/153); Bucaramanga and Sincelejo 3.27% each (5/153); Armenia, Barranquilla, and Pasto 2.61% each (4/153); Floridablanca and Popayán 1.96% each (3/153); Montería, Neiva, and Piedecuesta 1.31% each (2/153); and Chía, Chinchiná, Dosquebradas, Girardot, Ibagué, Magangué, Puerto Colombia, Santa Marta, Soledad, Valledupar, and Villavicencio only registered participation of 0.655 each (1/153) (Table 1).

### 3.2. Clinical Approach

**Figure 1:**
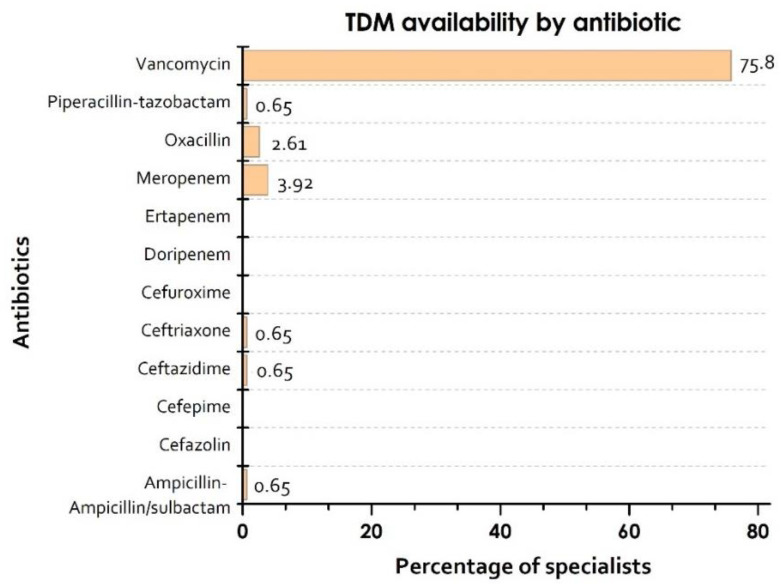
Antibiotics in which serum concentrations can be controlled according to availability in the hospital where the specialists work. TDM: therapeutic drug monitoring.

Of the specialists, 88.9% reported TDM availability and 21.57% made known access to results within 6 h. The main available antibiotic TDM was vancomycin (75.82%), with a low proportion of *β-lactams* TDM (Figure 1).

**Figure 2:**
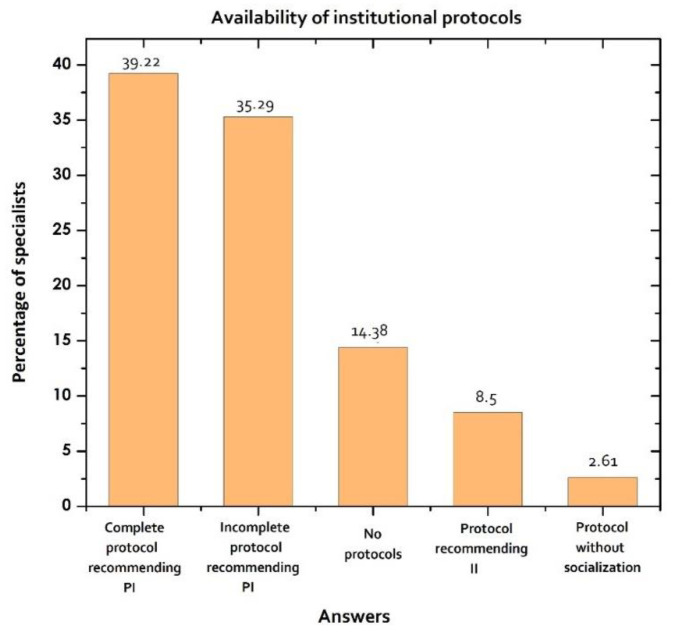
Characterization of the availability of institutional protocols to guide clinical practice. Complete protocols refer to those with additional information (product stability, dilution method and solution, maximum concentrations, duration of infusion, incompatibilities). PI: prolonged infusions, II: intermittent infusions.

Of the specialists, 22.2% reported pharmaceutical support, with a median of 3 visits per week of the infectious disease specialists. The results showed that 131/153 (85.62%) of the intensivists had some type of institutional protocol; however, only 39.22% had a complete and socialized protocol. Likewise, the specialists who have protocols in their institution but without information related to the stability of the drugs (incomplete protocols) represented 35.29% of the total of specialists surveyed (54/153) (Table 1). In 8.5% of the institutions where specialists work, they recommend the use of II in their protocols. No specialists considered institutional protocols to be unnecessary (0/153) and 2.61% reported that the existing protocols in their institution were not socialized to health professionals and were therefore unknown (Figure 2).

We found that 71.9% of the intensivists involved in the study preferred prolonged or continuous infusions. The median of the clinical cases approach score was 4.9/10, obtained based on the sum of the scores of each question regarding the loading dose and preference as to the use of PI in clinical cases and PK/PD targets. Only 10 people obtained a score greater than eight (the highest was 9.55). In regard to these questions, 33.33% of intensivists (51/153) reported the use of β-lactam loading doses for all types of infusions in cases of severe infections, compared to 20.26% (31/153) who said they never used them. The clinical case in which there were more incorrect answers was the prescription of vancomycin for bacteremia with MRSA and sepsis, at 72.54% (110/153), and the clinical case that had the most correct answers was the prescription of piperacillin-tazobactam for urinary tract infection by *Pseudomonas aeruginosa* and sepsis, with 78.43% (120/153). Regarding the objective value of the area under the curve over the minimum inhibitory concentration (AUC24/MIC) in critically ill patients with methicillin-resistant *Staphylococcus aureus* infections, only 54.24% (83/153) gave the correct answer (400−600 mg H/L). In regard to the last question, regarding the maximum number of hours (in optimal conditions) that an antibiotic is stable in an infusion solution, meropenem was the antibiotic on which the intensivists had the most knowledge, with 40.52% (62/153), and cefazolin was the one that they knew the least, at 11.11% (17/153).

### 3.3. Attitudes and Barriers towards Prolonged/Continuous Infusions and TDM

**Figure 3:**
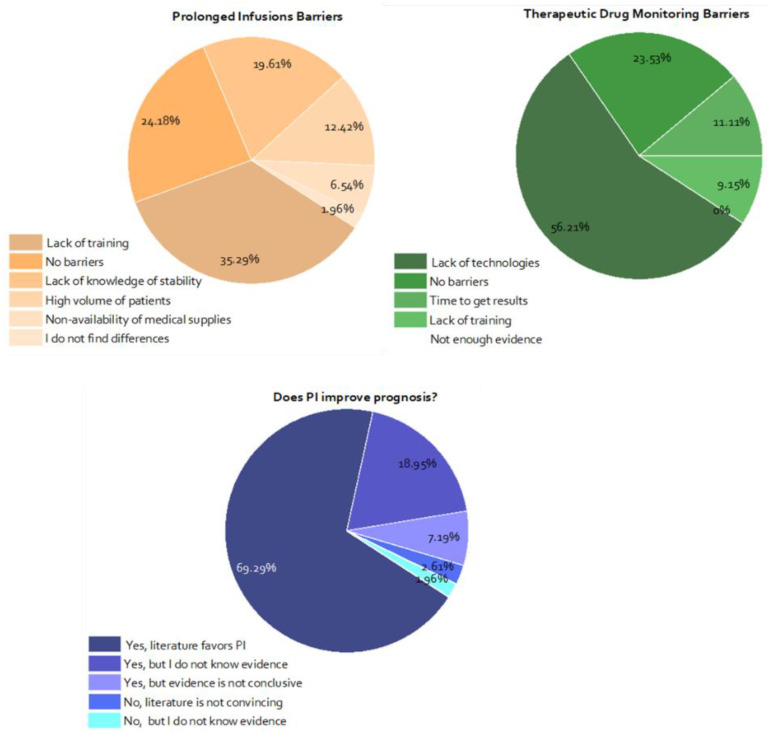
Perception of specialists on types of barriers related to prolonged infusions, TDM, and knowledge of the evidence. PI: prolonged infusions, II: intermittent infusions.

The main perceived barrier for choosing continuous or PI was the lack of training of personnel in this practice (35.29%). On the other hand, 56.21% of those surveyed considered that the lack of technologies for measuring antibiotic levels was the main barrier to performing TDM in their hospitals. Finally, most intensivists (69.28%) considered that the prolonged and continuous infusion of vancomycin and *β-lactams* improved the prognosis of patients with severe sepsis and that the scientific evidence pointed in this direction (Figure 3).

### 3.4. Factors Associated with the Use of Prolonged/Continuous Infusions

Regarding the bivariate approach, crude analysis suggested an association between the use of institutional protocols and TDM availability with the score obtained by the “approach to clinical cases” (*p*-value < 0.05) (Table 2). Initially, no association was found between sociodemographic and clinical practice variables with a preference for using continuous infusions or PIs (Table 3). Our findings suggest that clinical practice based on institutional protocols could explain the preference for using continuous infusions or PIs, as the odds of preferring continuous or extended infusions among those who did not have institutional protocols was 80% lower compared with those who had comprehensive protocols that recommend PI; OR 0.2 (CI95%: 0.06−0.61), *p*-value < 0.05. Similarly, the odds of using PI among those who had incomplete protocols was 65% lower than it was among those who had comprehensive protocols; OR 0.35 (CI95%: 0.14−0.87), *p*-value < 0.05.

## 4. Discussion

ANTIBIOCOL was the first nationwide survey evaluating the administration of prolonged and continuous infusions and drug monitoring of *β-lactams* and vancomycin in ICU settings in Colombia, representing an approach towards understanding the preferences and decisions made by critical care specialists in their daily practice. Its results could impact clinical practice, quality of care, and possibly the outcomes of critically ill patients within the framework of the antimicrobial stewardship strategies.

In our study, most of the participants were men between the ages of 21 and 40 in a 2:1 ratio compared to women, with informal education about antimicrobial stewardship, a median clinical experience of 10 years, and a monthly dedication of approximately 200 h, which demonstrated a high level of experience and workload. The origins of the surveyed physicians were diverse and included the main cities of Colombia, which shows that the random selection of the participants allowed the inclusion of different geographic territories and different levels of clinical experience.

Concerning administration practices, the majority of participants considered that a prolonged or continuous infusion of beta-lactam antibiotics and vancomycin improved the prognosis of their patients, and this is sustained by current evidence. This information is consistent with the proportion of physicians that use continuous infusion in Colombia (71.9%). Nevertheless, there are still many physicians who prefer bolus infusions over prolonged or continuous infusions, and this finding correlates with the lack of training of medical staff in this practice. We can find similar results in France, where 77% of the participants were convinced of the value of using continuous infusion for *β-lactams* and vancomycin above other administration methods [29]. Nonetheless, several studies in the United Kingdom evidenced a lower preference for this type of infusion (as low as 20%), although the evidence was in favor of its use, which places Colombia in an encouraging prospect [17,36,37].

Regarding TDM, we conclude that performing TDM for *β-lactams* remains an underused practice in Colombia, with the usage of TDM restricted to vancomycin and only in some cases to *β-lactams*, such as meropenem, piperacillin tazobactam, ampicillin sulbactam, and ceftazidime. Similar findings were obtained in other studies, especially in the only paper that included countries from Latin America [32]. Other studies have shown interesting results regarding antibiotic stewardship programs and TDM use, such as the study performed by Kim B et al., which demonstrated that, even in hospitals with antibiotic stewardship programs, TDM is still an underused practice, especially for *β-lactams* [38]. In our findings, a lack of technology was the main barrier reported by the participants. Since Colombia is still a developing country, the lack of technology in our hospitals is a common but concerning issue, leading physicians to work on the basis of the availability of supplies, rather than by following clinical guidelines.

Not only is the availability of TDM among the hospitals vital, so are the waiting times between sampling and the obtainment of results. Our study showed that results took more than 24 h to arrive in 30% of the reports. This should be considered another barrier to the use of TDM, since long waiting times to receive the results discourage clinicians from performing it; the main objective of TDM usage is to optimize dosing, meaning that results should be immediately available or at least take less than 24 h to enable decision-making as quickly as possible. Another obstacle found was the general knowledge gap regarding the use of continuous infusions in real clinical contexts, as well as PK/PD therapeutic targets and the stability of antibiotics, which could be explained by the lack of technology in many cases, because the evidence is recent and there are no clinical practice guidelines at a national level.

Even though conducting an analytical exploration was not the main objective, striking data were found. The main analytical result of this research indicates that the availability of complete institutional protocols with additional information significantly predicts the preference for continuous infusion and PI by intensivists. The availability of these protocols, even if they were incomplete, was above the values reported in other studies [28,29,30,31,32]. These results demonstrate the importance of having internal protocols in ICUs as a standardized method to improve clinical practices, such as choosing the right antibiotic, time, dosing, and the best method of administration. Furthermore, this confirms the need for unifying the national protocols to have an even more consistent clinical practice guidelines that could improve clinical outcomes.

This is a cross-sectional study that has limitations in determining causal association. In consequence, this research is an initial approach to describing practices and barriers, suggest possible action areas, and acting as the starting point for future research. Moreover, due to the absence of a list of every intensive care specialist in the country, the selection of the sample was made through the specialists registered in the AMCI, which is recognized as a limitation of this study. Additionally, non-significant results do not necessarily indicate a lack of association but may be due to the sample size that was initially calculated to obtain proportions (lack of statistical power). However, despite the above, the significant associations found reflect the strength of the association. Another strength of this study is the representativeness of the random sample that support the external validity of the study for Colombia, which included big and small cities, unlike similar studies from other countries that performed convenience sampling.

This study is the first of its type in a Latin American country and included the design of a survey adapted to the Colombian context and its needs, as well as addressing barriers pertaining to public health decision-making. In addition, this study can provide a starting point for further research regarding the correct methods used to perform TDM of *β-lactams*, vancomycin, and other antibiotic groups in the ICU setting. Likewise, future experimental or observational research should be carried out to assess clinical outcomes with the implementation of protocols involving continuous infusion and TDM practices, along with encouraging physicians to follow clinical guidelines or internal protocols to increase the accuracy of antimicrobial use among critically ill patients. Considering the barriers found among clinicians concerning the use of continuous or extended infusions, these should also be assessed in order to achieve higher rates of clinical cures and to reduce toxicity based on the available evidence on this practice.

## 5. Conclusions

This present paper is the first of its class in Colombia, involving a representative sample of intensive care specialists with vast clinical experience, who recognize continuous infusions as the preferred administration strategy to improve the prognosis of patients. Vancomycin was the most frequently monitored antibiotic and, for the most part, TDM for *β-lactams* was not available, which reflects a lack of relevant technologies in the country, recognized by specialists as the main barrier for its use. This technological gap not only affects the applicability of the evidence in the clinical context, but also impacts the degree of general knowledge about these practices. Other hurdles, such as scarce training and socialization of complete protocols, predict the possibility of choosing this type of administration for antibiotics. The findings described above may facilitate the development of interventions that influence these practices and the outcomes of critically ill patients, in favor of current antimicrobial stewardship strategies.

## Figures and Tables

**Table 1:** Descriptive analysis of sociodemographic information and clinical practice.

Descriptive Analysis				
Demographic	%	n/N	Median	IQR
Age (years)			41.50	(34.0−50.9)
≤30	12.42	19/153		
31−40	34.64	53/153		
41−50	28.76	44/153		
51−60	22.88	35/153		
≥60	1.31	2/153		
Sex				
Men	67.97	104/153		
Women	32.03	49/153		
University Hospital				
Yes	53.59	82/153		
No	46.41	71/153		
Type of ICU				
Medical	59.48	91/153		
Surgical	9.15	14/153		
Mixed	31.37	48/153		
Membership of the infection committee				
Yes	30.72	47/153		
No	69.28	106/153		
Experience time (years)			10	(4−17)
<5	30.07	46/153		
5−10	24.84	38/153		
>10	45.10	69/153		
Monthly dedication in ICU (Hours)			200	(160−250)
<60	9.15	14/153		
60−120	9.8	15/153		
121−200	32.68	50/153		
>200	48.37	74/153		
Antimicrobial education				
Formal	30.72	47/153		
Informal	49.67	76/153		
None of the Above	19.61	30/153		
Clinical practice	%	n/N	Median	IQR
Infectologist Weekly visits			3	(1−5)
0	7.19	11/153		
1–4	56.86	87/153		
5–7	35.95	55/153		
Institutional protocol				
Yes	85.62	131/153		
Complete protocol recommending PI	39.22	60/153		
Incomplete protocol recommending PI	35.29	54/153		
Protocol recommending II	8.5	13/153		
Protocol without socialization	2.61	4/153		
No	14.38	22/153		
No protocols	14.38	22/153		
Unnecessary	0	0/153		
TDM availability				
TDM not available	11.11	17/153		
<6 h	21.57	33/153		
6−12 h	28.10	43/153		
12−24 h	9.15	14/153		
24−48 h	13.73	21/153		
>48 h	16.34	25/153		
Pharmaceutical support				
Yes	22.22	34/153		
No	77.78	119/153		
Use of MIC				
Yes	75.82	116/153		
No	24.18	37/153		
Use of software				
Yes	26.80	41/153		
No	73.20	112/153		
Prolonged infusions				
Yes	71.90	110/153		
No	28.10	43/153		
Approach of clinical cases			4.9	(3.5−6.4)

PI: prolonged infusions, II: intermittent infusions.

**Table 2:** Bivariate analysis of approaches to clinical cases.

	Bivariate Analysis	
	Median (IQR)	*p*-value
Sex		
Male	5.0 (4.4, 6.4)	0.501
Female	4.9 (4.2, 6.4)	
University hospital		
Yes	4.9 (3.4, 6.4)	0.946
No	4.8 (4.3, 6.2)	
Membership of the infection committee		
Yes	4.7 (3.3, 6.6)	0.650
No	4.9 (4.2, 6.3)	
Antimicrobial education		
Formal education	4.9 (4.2, 6.4)	0.469
Informal education	4.8 (3.5, 6.4)	
None	4.8 (3.3, 5.9)	
Institutional protocol		
Complete protocol recommending PI	6 (4.4, 7.4)	0.032
Incomplete protocol recommending PI	4.7 (4.3, 6.1)	
Protocol recommending II	4.5 (3.3, 6.1)	
Protocol without socialization	4.8 (3.9, 5.2)	
No protocols	4.5 (3.0, 7.7)	
TDM availability		
TDM not available	4 (3.2, 4.9)	0.026
Vancomycin	5.1 (4.3, 6.5)	
*β-lactams* and vancomycin	4.6 (3.3, 5.7)	
Time to serum results		
TDM not available	4.5 (3.1, 5)	0.076
<6 h	5 (3.4, 7.2)	
6−12 h	4.6 (3.7, 6.2)	
12−24 h	5 (4.7, 6.2)	
24−48 h	5.7 (4.6, 6.4)	
> 48 h	4.9 (4.5, 6.4)	
Pharmaceutical support		
Yes	5.9 (3.4, 7.3)	0.424
No	4.8 (4.3, 6.2)	
Use of MIC		
Yes	4.9 (3.4, 6.4)	0.871
No	4.7 (4.3, 6.2)	
Use of software		
Yes	4.7 (3.3, 6.2)	0.273
No	4.9 (4.3, 6.4)	

PI: prolonged infusions, II: intermittent infusions.

**Table 3:** Bivariate analysis of preferences for the use of continuous or prolonged infusions.

	Bivariate Analysis		
	PI *n* = 110	II *n* = 43	*p*-value
Age, median years (IQR)			
	41.2 (34.1, 50.6)	45.4 (34.2, 55.6)	0.447
Sex, *n* (%)			
Male	76 (69)	28 (65.1)	0.778
Female	34 (30.9)	15 (34.9)	
University hospital, *n* (%)			
Yes	62 (56.4)	20 (46.5)	0.358
No	48 (43.6)	23 (53.5)	
Membership of the infection committee, *n* (%)			
Yes	35 (31.8)	12 (27.9)	0.782
No	75 (68.2)	31 (72.1)	
Experience time, median years (IQR)			
	10 (4, 16.7)	8 (3, 17.5)	0.205
Monthly dedication in ICU, median hours (IQR)			
	240 (160, 280)	200 (160, 240)	0.159
Antimicrobial education, *n* (%)			
Formal education	30 (27.3)	17 (39.5)	0.32
Informal education	58 (52.7)	18 (41.9)	
None	22 (20)	8 (18.6)	
Infectologist weekly visits, median days (IQR)			
	3 (1, 5)	3 (1, 7)	0.675
Institutional protocol, *n* (%)			
Complete protocol recommending PI	50 (45.5)	10 (23.3)	0.093
Incomplete protocol recommending PI	36 (32.7)	18 (41.9)	
Protocol recommending II	9 (8.2)	4 (9.3)	
Protocol without socialization	3 (2.7)	1 (2.3)	
No protocols	12 (10.9)	10 (23.2)	
TDM availability, *n* (%)			
TDM not available	23 (20.9)	15 (34.9)	0.102
Vancomycin	81 (73.6)	24 (55.8)	
*β-lactams* and vancomycin	6 (5.5)	4 (9.3)	
Time to serum results, *n* (%)			
TDM not available	20 (18.2)	13 (30.2)	0.538
<6 h	31 (28.2)	12(27.9)	
6−12 h	10 (9.1)	4 (9.3)	
12−24 h	15 (13.6)	6 (13.9)	
24−48 h	21 (19.1)	4 (9.3)	
>48 h	13 (11.8)	4 (9.3)	
Pharmaceutical support, *n* (%)			
Yes	27 (24.5)	7 (16.3)	0.373
No	83 (75.4)	36 (83.7)	
Use of MIC, *n* (%)			
Yes	83 (75.5)	33 (76.7)	1
No	27 (24.5)	10 (23.3)	
Use of software, *n* (%)			
Yes	28 (25.5)	13 (30.2)	0.691
No	82 (74.5)	30 (69.8)	

PI: prolonged infusions, II: intermittent infusions.

## Supplementary Materials

The following are available online at https://www.mdpi.com/article/10.3390/pharmaceutics13101577/s1, ANTIBIOCOL study questionnaire.
