# Supplementary material for: Administration and Therapeutic Drug Monitoring of β-lactams and Vancomycin in Critical Care Units in Colombia: The ANTIBIOCOL Study
Source: Pharmaceutics. 2021 Sep 28;13(10):1577. doi: 10.3390/pharmaceutics13101577 (PMC8537979; doi:10.3390/pharmaceutics13101577)
Supplement: Supplementary file 1 [file pharmaceutics-13-01577-s001.zip › pharmaceutics-1358085-supplementary-resub(1).docx]

Supplementary Materials: Administration and Therapeutic Drug Monitoring of B-lactams and Vancomycin in Critical Care Units in Colombia - The ANTIBIOCOL Study [1]

Yuli V. Fuentes, Jhosep Blanco, Diana Marcela Díaz-Quijano, Sharon Lechtig-Wasserman, Hans Liebisch-Rey, Nicolas Díaz-Pinilla, Peter Vergara-Ramirez and Rosa-Helena Bustos

**REGISTRY AND INFORMED CONSENT**

Name of principal investigator: Yuli Viviana Fuentes Barreiro, M.D. E-mail: [yulyfuba@unisabana.edu.co](mailto:yulyfuba@unisabana.edu.co)

Contact: Dr. Diana Díaz Telephone: +578615555 Ext.23313 E-mail**:** [**diana.diaz1@unisabana.edu.co**](mailto:diana.diaz1@unisabana.edu.co)

**The research groups Evidencia Terapéutica and Investigación en Salud of Universidad de La Sabana, hereby invite you to fill this questionnaire, as part of the 850th call for research of Colciencias and as the thesis of the master’s degree in Epidemiology. It seeks to describe administration practices and therapeutic monitoring of antibiotics –B-lactams and vancomycin- in intensive care units in Colombia. This survey is meant to be responded virtually, using Survey Monkey, and is directed towards specialists in adult intensive care medicine. The objective is to document clinical practice in the critical care units where you work most of the time.**

**Participation in this research is voluntary. With the authorization of the Colombian Society for Intensive Care Medicine (AMCI) and according to the ethic guidelines (R008430 de 1993) of the Ministry of Health and Social Protection, the information that you supply, will no result or end in any risk, and filling it implies your consent. This questionnaire will be confidential and data protection will be guaranteed (Habeas Data – Law 1581/2012 and Dec 1377/2013 MinTIC). Furthermore, participants will be allowed to leaved the study freely at anytime by contacting the investigators by whichever of the provided means. Once the results of the research are obtained, the researchers pledge to socialize them with the participants and with interested members of the scientific community.**

**To encourage participation in this research project, a raffle of 3 (three) gift cards with a value of $150.000 COP in selected brands will be performed, for those participants who fill the entire questionnaire. To access the terms and conditions of the raffle, click on the following hyperlink.**

**Approximate duration: 5 to 10 minutes.**

**Questionnaire open until March 31^st^ 2021 (24:00 hours).**

**Do you accept to participate in this research by responding to the following questionnaire?**

1. **Yes**
2. **No**

**If you do not accept, send the questionnaire without responding.**

**Register the e-mail with which you are associated to AMCI (remember, this is the only way we can identify you as a participant for the final raffle):**

**________________**

**Confirmation of the e-mail with which you are associated to AMCI**

**________________**

**Select the department and city where you work most of the time (display options).**

**Part 1: sociodemographic information**

1. Date of birth

DD/MM/YYYY

1. Gender:
2. Male
3. Female
4. Prefer not to say
5. Does the intensive care unit where you work most of the time form part of a university hospital?
6. Yes
7. No
8. In what type of intensive care unit do you work most of the time?
9. Surgical intensive care unit
10. Mixed intensive care unit
11. Medical intensive care unit
12. Do you belong to any of the committees related to vigilance and prescription of antimicrobial drugs (epidemiology, infection committee, others)?
13. Yes
14. No
15. Time in years of experience in intensive care units:

___________

1. How many hours a month do you work in intensive care units?

___________

1. Have you received education in the prescription or monitoring of antimicrobial drugs?
2. Yes, formal education (specializations or master degrees)
3. Yes, informal education (courses, trainings, others)
4. None of the above

**Part 2: clinical practice**

Answer the following questions according to what happens in the intensive care unit where you work most of the time.

1. How many times a week does an infectious disease specialist go to your intensive care unit for consult and to provide advice?
2. We do not have an infectious disease specialist
3. Once a week
4. Twice a week
5. Three times per week
6. Four times per week
7. Five times per week
8. Six times per week
9. Seven times per week
10. Do you have institutional protocols to guide the administration of B-lactams and vancomycin?
11. Yes, there is a protocol at my hospital that recommends the continuous infusion of these antibiotics and I have additional information (stability of the product, mode and solution of dilution, maximum concentrations, infusion duration, incompatibilities)
12. Yes, there is a protocol at my hospital that recommends the continuous infusion of these antibiotics, but I DO NOT have additional information (stability of the product, mode and solution of dilution, maximum concentrations, infusion duration, incompatibilities)
13. Yes, there is a protocol at my hospital, and it recommends the administration of fluid boluses of these antibiotics
14. Yes, there is a protocol at my hospital, but it has not been disclosed and I do not know its recommendations
15. No, but this information would be useful for me
16. No, but I do not require this information
17. Which serum concentrations of antibiotics can be controlled at your hospital (therapeutic drug monitoring = TDM)? (You can select more than one option).
18. TDM not available at my institution
19. Oxacillin
20. Ampicillin – Ampicillin/sulbactam
21. Cefazolin
22. Cefuroxime
23. Ceftriaxone
24. Ceftazidime
25. Cefepime
26. Piperacillin-tazobactam
27. Meropenem
28. Ertapenem
29. Doripenem
30. Vancomycin
31. ¿How much does it take for you to receive the result of the serum levels of antibiotics at your institution?
32. Does not apply (TDM not available)
33. <6 hours after taking the blood sample
34. 6-12 hours
35. 12-24 hours
36. 24-48 hours
37. >48 hours
38. Does a pharmacist/pharmacologist or other specialist usually contact you to assist in the interpretation of the result?
39. Yes
40. No
41. Do you usually receive the MIC of isolated bacteria in your institution?
42. Yes
43. No
44. Do you use any software to adjust antibiotic doses administered to your patients?
45. Yes
46. No

**Part 3: clinical knowledge**

*In the following clinical bullet points, intermittent infusion corresponds to infusion during <30 minutes, prolonged infusion to infusions that last 2-4 hours and continuous infusion to infusion with a syringe pump or its equivalent (changing the syringe every 8-12 hours, if necessary).*

***The objective of this part of the questionnaire is to evaluate daily practices for these types of infusion through your answers in the clinical cases.***

1. Is a loading dose usually given for B-lactams in serious infections?
2. *Yes, for intermittent infusions*
3. *Yes, for prolonged infusions*
4. *Yes, for continuous infusions*
5. *Yes, for all types of infusions*
6. *Never*

*In the following cases, you are not asked to indicate the loading dose. All the cases describe an adult with severe sepsis, with a weight of 80 kg, and a normal renal function.*

1. Case 1: how is piperacillin-tazobactam usually prescribed for urinary tract infections by Pseudomonas aeruginosa and sepsis?
2. Intermittent infusion, 4 infusions lasting 30 minutes each
3. Prolonged infusion, 4 infusions lasting 2-4 hours each
4. Continuous infusion
5. Case 2: how do you usually prescribe cefepime o meropenem for pneumonia by P. aeruginosa, in a patient with COPD and sepsis?
6. Intermittent infusion, 3 infusions lasting 30 minutes each
7. Prolonged infusion, 3 infusions lasting 2-4 hours each
8. Continuous infusion
9. Case 3: how is ceftriaxone usually prescribed for meningitis by Streptococcus pneumoniae and sepsis?
10. Intermittent infusion, 2 infusions lasting 30 minutes each
11. Prolonged infusion, 2 infusions lasting 2-4 hours each
12. Continuous infusion
13. How is vancomycin for bacteremia by MRSA and sepsis usually prescribed?
14. Intermittent infusion, 2 infusions lasting 60 minutes each
15. Continuous infusion
16. Which is the goal value of the area under the curve over the minimum inhibitory concentration (AUC_24_/MIC), in the critically ill patient with infections by methicillin resistant Staphylococcus aureus?
17. 200-400 mg·h/L
18. 300-500 mg·h/L
19. 400-600 mg·h/L
20. 600-800 mg·h/L
21. In your opinion, what is the maximum number of hours (in ideal conditions)^[[1]](#footnote-1)^ that the following antibiotics are stable for, in an infusion solution?
22. Oxacillin
23. Ampicillin- Ampicillin/sulbactam
24. Cefazolin
25. Cefuroxime
26. Ceftriaxone
27. Ceftazidime
28. Cefepime
29. Piperacillin-tazobactam
30. Meropenem
31. Vancomycin

Proposed answers for each one:

<2 hours

2-4 hours

4-12 hours

>12 hours

**Part 4: perception**

1. What do you think is the main obstacle at your intensive care unit for the administration of continuous infusions of antimicrobial drugs?
2. Large number of patients that prevents monitoring of the continuous infusion
3. Insufficient amount of continuous infusion pumps
4. Not knowing the stability of the drug
5. Lack of training of personnel in this practice
6. I don’t see any differences between the administration of boluses vs continuous infusion
7. There are no obstacles
8. What do you think is the main obstacle at your intensive care unit for the measurement of therapeutic levels of B-lactams and/or vancomycin?
9. Personnel not trained in this practice
10. Lack of technologies to perform it
11. The time between the moment the sample is taken and the result is obtained
12. I consider that there is insufficient scientific evidence to perform this practice
13. There are no obstacles
14. Do you think that the prolonged/continuous infusion of B-lactams and vancomycin improve the prognosis of patients with severe sepsis?
15. Yes, and the scientific literature points in this direction
16. Yes, but I do not know the literature on this topic
17. Yes, but the literature still is not clear on this topic
18. No, because the current literature is not convincing
19. No, but I am not familiar with the literature on this topic

Optimal conditions at 25°C

**CORRECT ANSWERS OF PART 3:**

**CLINICAL KNOWLEDGE TEST FOR “APPROACH TO CLINICAL CASES” VARIABLE [2]**

16. Is a loading dose usually given for B-lactams in serious infections?

d. Yes, for all types of infusions

17. Case 1: how is piperacillin-tazobactam usually prescribed for urinary tract infections by Pseudomonas aeruginosa and sepsis?

b. Prolonged infusion, 4 infusions lasting 2-4 hours each

or

c. Continuous infusion

18. Case 2: how do you usually prescribe cefepime o meropenem for pneumonia by *P. aeruginosa*, in a patient with COPD and sepsis?

b. Prolonged infusion, 3 infusions lasting 2-4 hours each

or

c. Continuous infusion

19. Case 3: how is ceftriaxone usually prescribed for meningitis by Streptococcus pneumoniae and sepsis?

a. Intermittent infusion, 2 infusions lasting 30 minutes each

20. How is vancomycin for bacteremia by MRSA and sepsis usually prescribed?

b. Continuous infusion

21. Which is the goal value of the area under the curve over the minimum inhibitory concentration (AUC24/MIC), in the critically ill patient with infections by methicillin resistant Staphylococcus aureus?

c. 400-600 mg·h/L [3]

22. In your opinion, what is the maximum number of hours (in ideal conditions) that the following antibiotics are stable for, in an infusion solution? [2]

a. Oxacillin: > 12 hours

b. Ampicillin- Ampicillin/sulbactam: 4-12 hours

c. Cefazolin: > 12 hours

d. Cefuroxime: > 12 hours

e. Ceftriaxone: <2 hours

f. Ceftazidime: 4-12 hours

g. Cefepime: 4-12 hours

h. Piperacillin-tazobactam: > 12 hours

i. Meropenem: 4-12 hours

j. Vancomycin: > 12 hours

**References**

1. Charmillon, A.; Novy, E.; Agrinier, N.; Leone, M.; Kimmoun, A.; Levy, B.; Demore, B.; Dellamonica, J.; Pulcini, C. The ANTIBIOPERF study: a nationwide cross-sectional survey about practices for beta-lactam administration and therapeutic drug monitoring among critically ill patients in France. *Clin Microbiol Infect.* **2016**, *22*, 625-631.

2. Longuet, P.; Lecapitaine, A.L.; Cassard, B.; Batista, R.; Gauzit, R.; Lesprit, P.; Haddad, R.; Vanjak, D.; Diamantis, S. Preparing and administering injectable antibiotics: How to avoid playing God. *Med Mal Infect.* **2016**, *46*, 242-268.

3. Rybak, M.J.; Le, J.; Lodise, T.P.; Levine, D.P.; Bradley, J.S.; Liu, C.; Mueller, B.A.; Pai, M.P.; Wong-Beringer, A.; Rotschafer, J.C., et al. Therapeutic monitoring of vancomycin for serious methicillin-resistant Staphylococcus aureus infections: A revised consensus guideline and review by the American Society of Health-System Pharmacists, the Infectious Diseases Society of America, the Pediatric Infectious Diseases Society, and the Society of Infectious Diseases Pharmacists. *American journal of health-system pharmacy : AJHP : official journal of the American Society of Health-System Pharmacists.* **2020**, *77*, 835-864.

1. Optimal conditions at 25°C [↑](#footnote-ref-1)
